# Insulin facilitates entry of calcium ions into human and murine erythrocytes via Piezo1: a newly identified mechanism with implications for type 2 diabetes

**DOI:** 10.1111/febs.70157

**Published:** 2025-06-08

**Authors:** Lennart Kuck, Tia A. Griffith, Antony P. McNamee, Jason N. Peart, John H. Wilson, Ajay Sharma, Lavanya A. Sharma, Kai Robertson, Eugene F. Du Toit, Michael J. Simmonds

**Affiliations:** ^1^ Biorheology Research Laboratory Griffith University Gold Coast Australia; ^2^ School of Pharmacy and Medical Science Griffith University Gold Coast Australia; ^3^ School of Medicine and Dentistry Griffith University Gold Coast Australia

**Keywords:** calcium signaling, nitric oxide, Piezo1, red blood cells, type 2 diabetes

## Abstract

Circulatory deficits are common and pathophysiologically relevant in type 2 diabetes mellitus (T2DM). Perturbed red blood cell (RBC) homeostasis and diminished nitric oxide (NO) availability contribute to endothelial dysfunction, a hallmark of cardiometabolic disorders; however, underlying pathophysiological mechanisms remain elusive. Here, we investigated RBC signaling pathways in a murine model of metabolic disease, focused on NO. T2DM‐RBCs had elevated levels of cytosolic NO, intracellular calcium ions (Ca^2+^), and reactive oxygen species. Acute stimulation with exogenous insulin had no effect on NO content. Whereas insulin exposure caused Ca^2+^ entry into healthy RBCs, T2DM‐RBCs were insensitive. Using RBCs isolated from human blood, we confirmed that insulin had no effect on RBC‐NO, despite prompting Ca^2+^ uptake. Ca^2+^ uptake with insulin exposure was sensitive to inhibition of mechanosensitive ion channels, as well as Ca^2+^ chelation. Furthermore, co‐incubation of RBCs with the piezo‐type mechanosensitive ion channel component 1 (Piezo1) channel agonist Yoda1 and insulin did not produce compounded Ca^2+^ uptake, raising the possibility of crosstalk between insulin and Piezo1. The hyperinsulinemia associated with T2DM may exacerbate normal Piezo1‐dependent Ca^2+^ uptake into RBCs, contributing to RBC dysfunction and circulatory complications in T2DM. The significance of RBC signaling in the pathophysiology of cardiometabolic disorders is still emerging. Individuals carrying mutations in the *PIEZO1* gene exhibit hematological aberrations and hereditary anemia, supporting the importance of Piezo1 in RBC homeostasis. Furthermore, a shift in RBC–NO metabolism favoring nitrosative stress may contribute to circulatory complications observed in metabolic diseases such as T2DM. Collectively, the emerging relevance of RBC signaling pathways may provide novel avenues for targeted drug development.

## Graphical abstract

Despite being enucleated, mammalian red blood cells (RBCs) possess complex signaling mechanisms. Here, we show that insulin signals through the mechanosensitive channel Piezo1 to promote influx of calcium ions. This newly identified pathway is disrupted in a murine model of type 2 diabetes and may contribute to driving circulatory deficits in prevalent metabolic disorders, a finding that may provide new therapeutic opportunities.

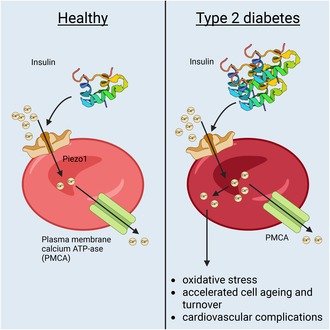

**Abbreviations**
Ca^2+^
: calcium‐ions
DAF‐FM: 4‐amino‐5‐methylamino‐2′,7′‐difluorofluorescein
EGTA: ethylene glycol‐bis(β‐aminoethyl ether)‐N,N,N′,N′‐tetraacetic acid
EI: elongation index
GTT: glucose tolerance test
HFD: high fat diet
*L*‐NAME: N(ω)‐nitro‐L‐arginine methyl ester
MAf: microcytic anemia factor
MCV: mean cell volume
NO: Nitric oxide
RDW: red cell distribution width
RMSD: root mean square deviation
ROS: reactive oxygen species
SNP: sodium nitroprusside
SS: shear stress
STZ: streptozotocin
T2DM: type 2 diabetes mellitus

## Introduction

Type 2 diabetes mellitus (T2DM) is a metabolic disorder characterized by chronically elevated blood glucose levels, often accompanied by hyperinsulinemia, chronic inflammation, hypertension, and oxidative stress [1]. Individuals with advanced T2DM tend to exhibit insulin resistance and may require exogenous insulin to support glucose clearance [2]. Up to 80% of morbidity and mortality in T2DM can be attributed to neuro‐ and cardiovascular complications [3, 4]. These complications appear diverse, although poor tissue perfusion and vascular dysfunction are hallmarks of T2DM [5], and could precipitate multi‐organ damage. Classically, processes that interfere with normal vascular function and vasodilation, including persistent inflammation, insulin resistance, and the production of free radicals, are considered primary pathophysiological mechanisms of T2DM [6, 7, 8, 9]. Research efforts in this field have therefore focused on vascular health in addition to managing glycemic control; however, there is evidence of a newly discovered source of vasomodulators that may also contribute to the development of endothelial dysfunction and circulatory deficits in T2DM: red blood cells (RBC; [10]).

While the primary source of vital vasodilatory compounds, such as the highly reactive signaling molecule nitric oxide (NO), is the endothelium [11], it is increasingly recognized that RBC are independent contributors to the circulating vasodilator pool [12]. While NO production appears to occur following acute stimulation with insulin in RBC [13] and endothelial cells [14], the impact of chronic hyperinsulinemia—as is often present in T2DM—on RBC‐derived NO is not known. Zhou *et al*. [15] convincingly demonstrate that RBC obtained from patients with T2DM induce endothelial dysfunction in previously healthy arterial tissue *ex vivo* via a reactive oxygen species (ROS)‐dependent mechanism. Further evidence supports both abnormal vascular signaling [16] and dysfunctional redox status of RBC [17] as potential mechanisms underpinning the proposed role of RBC in contributing to cardiovascular complications in T2DM. Collectively, hallmark pathophysiological characteristics of T2DM, including endothelial dysfunction and poor tissue perfusion, may be precipitated by dysfunctional RBC‐dependent regulation of vascular tone. Given RBC have only recently come into focus as potential drivers of T2DM pathogenesis, the underlying mechanisms are poorly understood [10].

Potential paracrine signaling effects of RBC‐generated NO are contentious [18]; nevertheless, evidence suggests that RBC‐NO regulates systemic blood pressure independent of the endothelium [19]. Hallmarks of T2DM‐related cardiovascular complications include hypertension and endothelial dysfunction, which strongly depend on NO bioavailability [5]. Given that RBC with abnormal NO metabolism obtained from individuals with T2DM induce endothelial dysfunction [17], it is plausible to hypothesize that pathologically altered RBC signaling would contribute to circulatory impairments in T2DM. Further, increased cytosolic calcium‐ion concentration [Ca^2+^]_i_ appears to be a prerequisite for RBC‐NO production [12, 20] and has been reported in individuals with T2DM [21]; indeed, the mechanosensitive cation channel Piezo1 may act upstream of NO in both endothelial cells [22, 23] and RBC [20]. The interplay between insulin‐ and Ca^2+^‐dependent stimulation of RBC‐NO production remains unclear, and it is currently unknown whether this signaling axis is pathologically altered in the T2DM environment.

The aim of the present study was therefore to assess whether NO‐ and/or Ca^2+^‐dependent RBC signaling pathways are altered in a murine diet‐induced model of T2DM. Further, given that insulin acts as a critical signaling molecule participating in the regulation of endothelial‐ and RBC‐derived vasodilators [13, 14, 24], a secondary aim was to investigate whether NO‐ and/or Ca^2+^‐dependent RBC signaling pathways were sensitive to acute exposure to exogenous insulin.

The main findings of this study indicate that the cytosolic concentration of NO and Ca^2+^ is pathologically increased in RBC from T2DM mice. Further, we reveal a novel signaling pathway in RBC, wherein insulin facilitates Ca^2+^‐uptake via the mechanosensitive cation channel Piezo1, likely through a secondary signaling pathway rather than a direct interaction. This mechanism appears to be dysfunctional in T2DM, which may explain some of the microcirculatory complications observed in T2DM.

## Results

### Characterization of blood obtained from the T2DM murine model generated via STZ injection combined with feeding a high‐fat diet

**Fig. 1:**
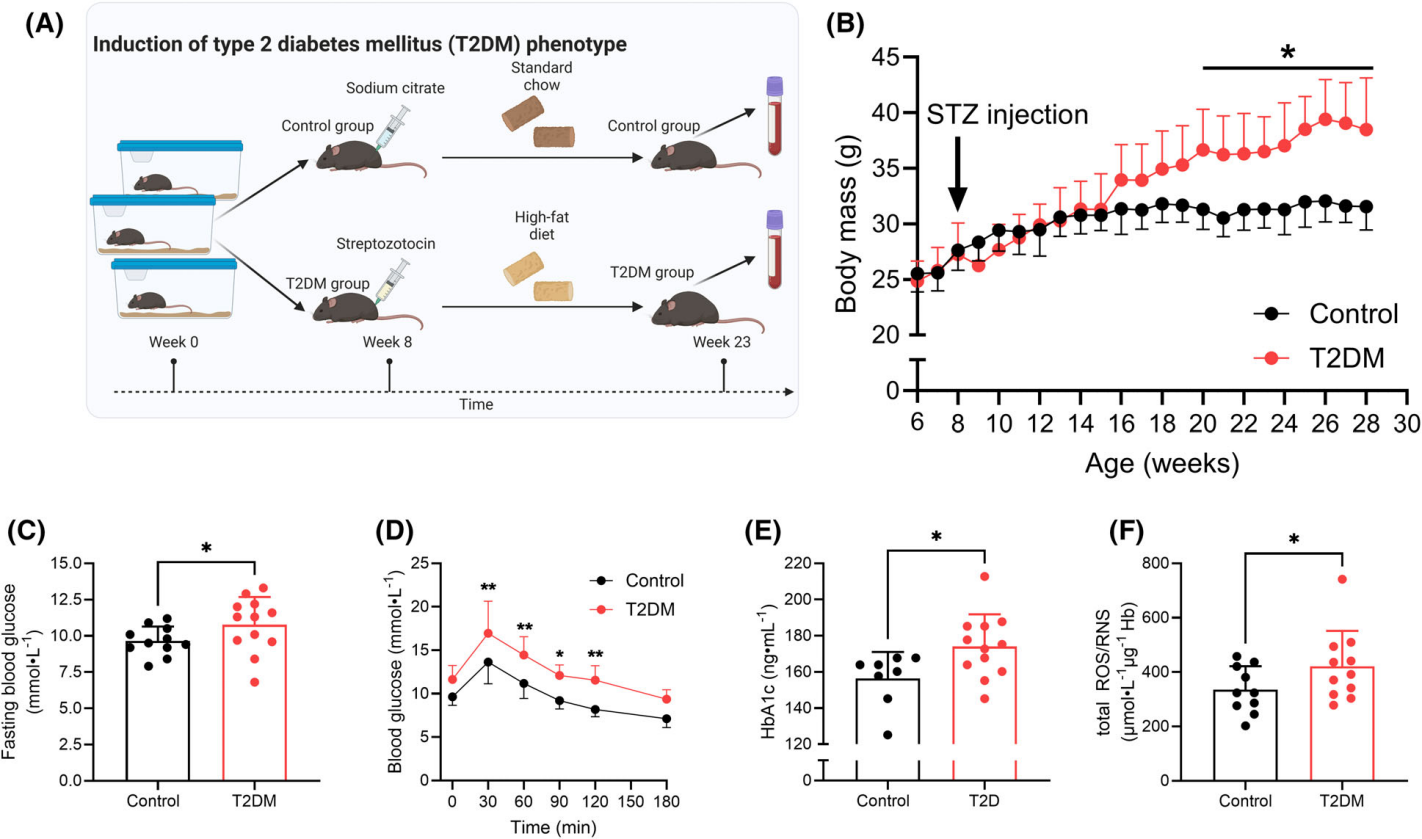
Injection of mice with streptozotocin followed by feeding of a high‐fat diet induced a type 2 diabetes mellitus‐like phenotype. (A) Mice were injected with streptozotocin (STZ) and then fed a high‐fat diet for 15 weeks to produce a type 2 diabetes mellitus‐like phenotype (T2DM). (B) Body mass increased significantly in T2DM mice after 15 weeks, while control mice remained stable. (C) Fasting blood glucose concentration was significantly elevated in T2DM mice when compared with control mice. (D) Glucose tolerance was impaired as indicated by elevated blood glucose concentration at 30 to 120 min during the glucose tolerance test. (E, F) Concentration of glycated hemoglobin and reactive oxygen species was increased with T2DM. *P < 0.05, **P < 0.01 determined using two‐way ANOVA (B, D) or unpaired t‐tests (C, E, F). Control *n* = 7–11 biological replicates (blood samples obtained from distinct donors); T2DM *n* = 8–11 biological replicates (blood samples obtained from distinct donors). Error bars denote standard deviation.

A combination of STZ injection and feeding of a high‐fat diet induces a T2DM phenotype in mice [25], which we sought to confirm for the present model (Fig. 1A). Body mass significantly increased in T2DM mice from 12 week post‐STZ injection and remained elevated by ~22% at 20 week when compared with control (*P* < 0.05; Fig. 1B). Fasting blood glucose at 20 week was significantly elevated by ~13% (*P* < 0.05; Fig. 1C) in T2DM mice when compared with control. Further, glucose clearance capacity was significantly impaired in T2DM mice when compared with control animals (*P* < 0.05; Fig. 1D), while glycated hemoglobin concentration was increased by ~11% (*P* < 0.05; Fig. 1E), collectively indicating that the present model exhibited a T2DM phenotype. The concentration of total reactive oxygen and nitrogen species (ROS/RNS) in RBC obtained from T2DM mice was significantly elevated by ~26% when compared to control (*P* < 0.05; Fig. 1F).

### Hematological parameters but not erythrocyte mechanics are impaired in the T2DM murine model

**Fig. 2:**
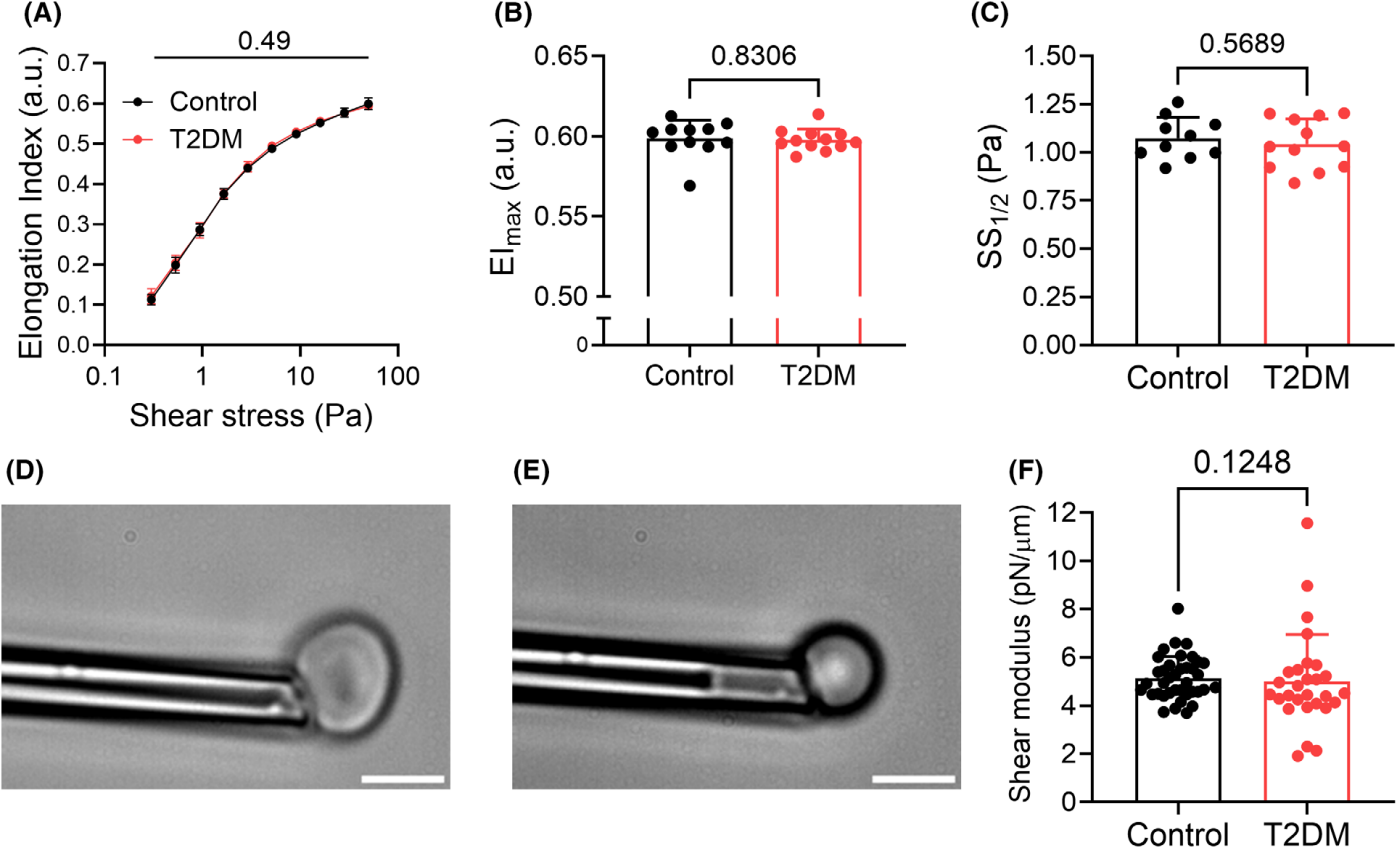
Murine erythrocyte mechanics in a type 2 diabetes mellitus‐like phaenotype model. (A) Elongation index (reflective of red cell deformability), measured across a range of shear stresses between 0.3–50 Pa, was unaltered. (B, C) Maximal deformability (EI_max_) and shear stress required for half‐maximal deformation (SS_1/2_) of red blood cells (RBC) from control mice was not different from that of RBC obtained from T2DM mice. (D–F) Shear modulus of individual RBC was measured by aspiration into a micropipette with an inner diameter of 1 μm. Single‐cell data from 1–3 mice were pooled (*n* = 28–38 individual RBC). Control *n* = 7–11 biological replicates (blood samples obtained from distinct donors); T2DM *n* = 8–11 biological replicates (blood samples obtained from distinct donors). Statistical significance determined using two‐way ANOVA (A) or unpaired t‐tests (B, C, F). Scale bars = 5 μm. Error bars denote standard deviation.

No significant differences in RBC deformability could be detected between control and T2DM mice at a cell population level (Fig. 2A–C). Membrane shear modulus, obtained at single‐cell resolution, indicates that there were no differences in mean membrane deformability of the RBC population (5.1 vs 5.0 pN·μm^−1^; Fig. 2D–F); however, the RBC population obtained from T2DM mice showed greater variability (standard deviation = 0.9 vs 1.9 pN·μm^−1^).

Induction of the T2DM phenotype in mice caused significant changes in hematological parameters. White blood cell count increased, along with RBC count (Table 1). Hemoglobin, hematocrit, mean cell volume, and mean corpuscular hemoglobin were all increased in mice with T2DM when compared with controls (Table 1). Mean cell hemoglobin concentration, red cell distribution width (RDW), and RDW‐standard deviation, however, were not significantly different between the groups (Table 1). Platelet counts were significantly elevated in T2DM mice, and mean platelet volume tended to be lower in mice with induced T2DM, albeit no statistically significant effect was detected (*P* = 0.053; Table 1). Moreover, the microcytic anemia factor was significantly increased in mice with T2DM when compared with control (Table 1).

**Table 1:** Complete blood count of blood samples obtained from healthy mice (Control) and mice with type 2 diabetes mellitus phenotype induced by streptozotocin and high‐fat diet (T2DM). Data presented as mean ± standard deviation. **P < 0.01, *P < 0.05, determined using unpaired t‐tests. Control *n* = 8; T2DM *n* = 11.

Complete blood count units	Control	Type 2 diabetes mellitus (T2DM)
White blood cells (×10^9^)	2.21 ± 0.96	4.73 ± 1.89**
Platelets (×10^9^)	66.65 ± 37.96	153.56 ± 116.35*
Red blood cells (×10^9^)	4.89 ± 1.22	6.35 ± 1.40*
Hemoglobin (g·dL^−1^)	85.79 ± 22.45	114.32 ± 25.42*
Hematocrit (L·L^−1^)	0.23 ± 0.06	0.30 ± 0.07*
Mean corpuscular volume (fL)	46.11 ± 0.88	47.39 ± 1.01*
Mean corpuscular hemoglobin (pg)	17.50 ± 0.35	18.01 ± 0.48*
Mean corpuscular hemoglobin concentration (g·dL^−1^)	375.25 ± 14.68	379.82 ± 8.44
Mean platelet volume (fL)	11.46 ± 1.74	9.62 ± 2.14
Red cell distribution width (%)	22.58 ± 2.51	21.12 ± 1.20
Microcytic anemia factor (a.u.)	4.00 ± 1.05	5.44 ± 1.25*

### Erythrocytes obtained from mice with T2DM exhibit elevated nitric oxide content and cytosolic calcium‐ion concentration

Following characterization of biophysical properties, we then assessed biochemical RBC properties, with an emphasis on NO metabolism and Ca^2+^.

**Fig. 3:**
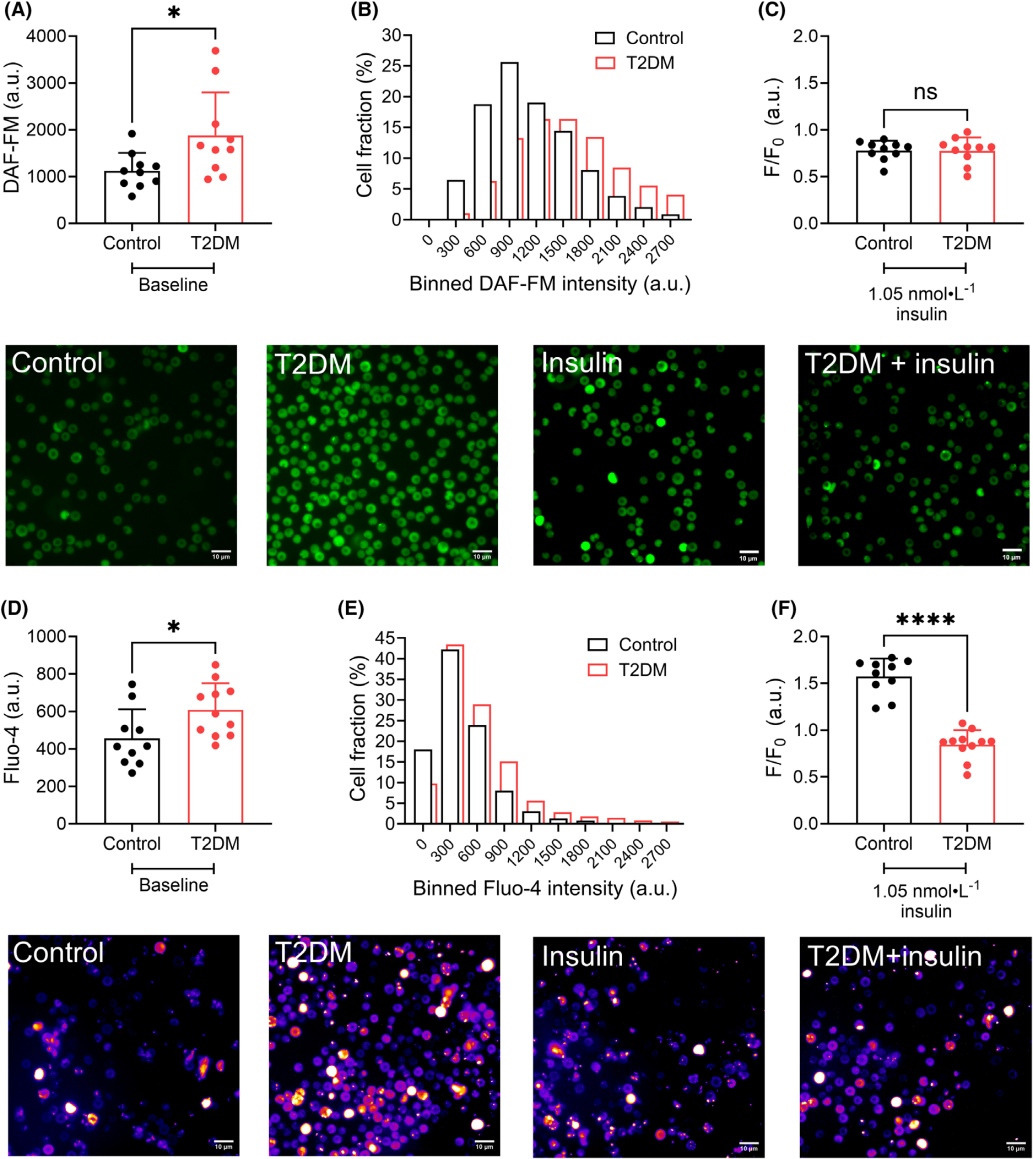
Murine erythrocyte nitric oxide and calcium‐ion signaling in a type 2 diabetes mellitus‐like phenotype model. (A, B) Mean fluorescent intensity (MFI) and single‐cell fluorescent intensity of RBC obtained from healthy control mice and mice with type 2 diabetes mellitus‐like phenotype (T2DM) incubated with the nitric oxide probe DAF‐FM are shown in absolute units. (C) Changes in MFI in response to insulin exposure (1.05 nmol·L^−1^) are shown relative to the baseline signal of each individual sample. Representative micrographs of DAF‐FM loaded RBC relating to the data presented in A–C are shown in green. (D, E) Basal MFI and single‐cell fluorescent intensity of RBC obtained from healthy control mice and mice with T2DM phenotype incubated with the calcium probe Fluo‐4 is shown in absolute units. (F) Changes in MFI in response to insulin exposure (1.05 nmol·L^−1^) are shown relative to the baseline signal of the same samples. Representative micrographs of Fluo‐4 loaded RBC relating to the data presented in D–F are shown in blue/red. Control *n* = 10 biological replicates (blood samples obtained from distinct donors); T2DM *n* = 10–11 biological replicates (blood samples obtained from distinct donors). A total of 150–300 individual RBC per sample were analyzed. *P < 0.05, ****P < 0.0001 determined using unpaired (A, C) or paired (D, F) t‐tests. Scale bars = 10 μm. Error bars denote standard deviation.

Isolated RBC from control mice exhibited significantly lower basal DAF‐FM fluorescent intensity when compared with those obtained from T2DM mice (*P* < 0.05; Fig. 3A). While ~25% of all control RBC exhibited a fluorescent signal of <900, in the T2DM RBC population it was less than ~9% (Fig. 3B). Upon exposure of control and T2DM DAF‐loaded RBC to insulin, no relative increase in fluorescent intensity was observed, irrespective of the presence of T2DM (*P* = 0.95; Fig. 3C).

Basal Fluo‐4 fluorescence intensity, which is reflective of [Ca^2+^]_i_, was significantly increased ~1.33‐fold in RBC obtained from T2DM mice when compared with RBC from healthy control mice (*P <* 0.05; Fig. 3D). In the control RBC population, ~41% of cells exhibited fluorescence of ≤200, while in the T2DM RBC population it was only 28% (Fig. 3E). When acutely exposed to insulin, Fluo‐4 fluorescence of control RBC significantly increased by ~1.57‐fold (*P <* 0.0001; Fig. 3F), while fluorescence of RBC obtained from T2DM mice remained at basal levels.

Insulin‐dependent NO production is well established in endothelial cells as a Ca^2+^‐independent mechanism [14], which has also been reported for human RBC [13, 24]. Our data indicated that while insulin stimulation did not affect RBC‐NO, it appears that RBC from healthy mice may take up Ca^2+^ in response to acute insulin stimulation, while RBC from T2DM were unresponsive to insulin. We thus sought to confirm whether this insensitivity of RBC‐NO was unique to murine RBC, and further, we aimed to assess whether insulin‐dependent Ca^2+^ uptake also occurs in RBC from healthy humans and interrogate the potential involvement of mechanosensitive cation channels in this response.

### Insulin has no effect on nitric oxide in human erythrocytes from healthy donors

**Fig. 4:**
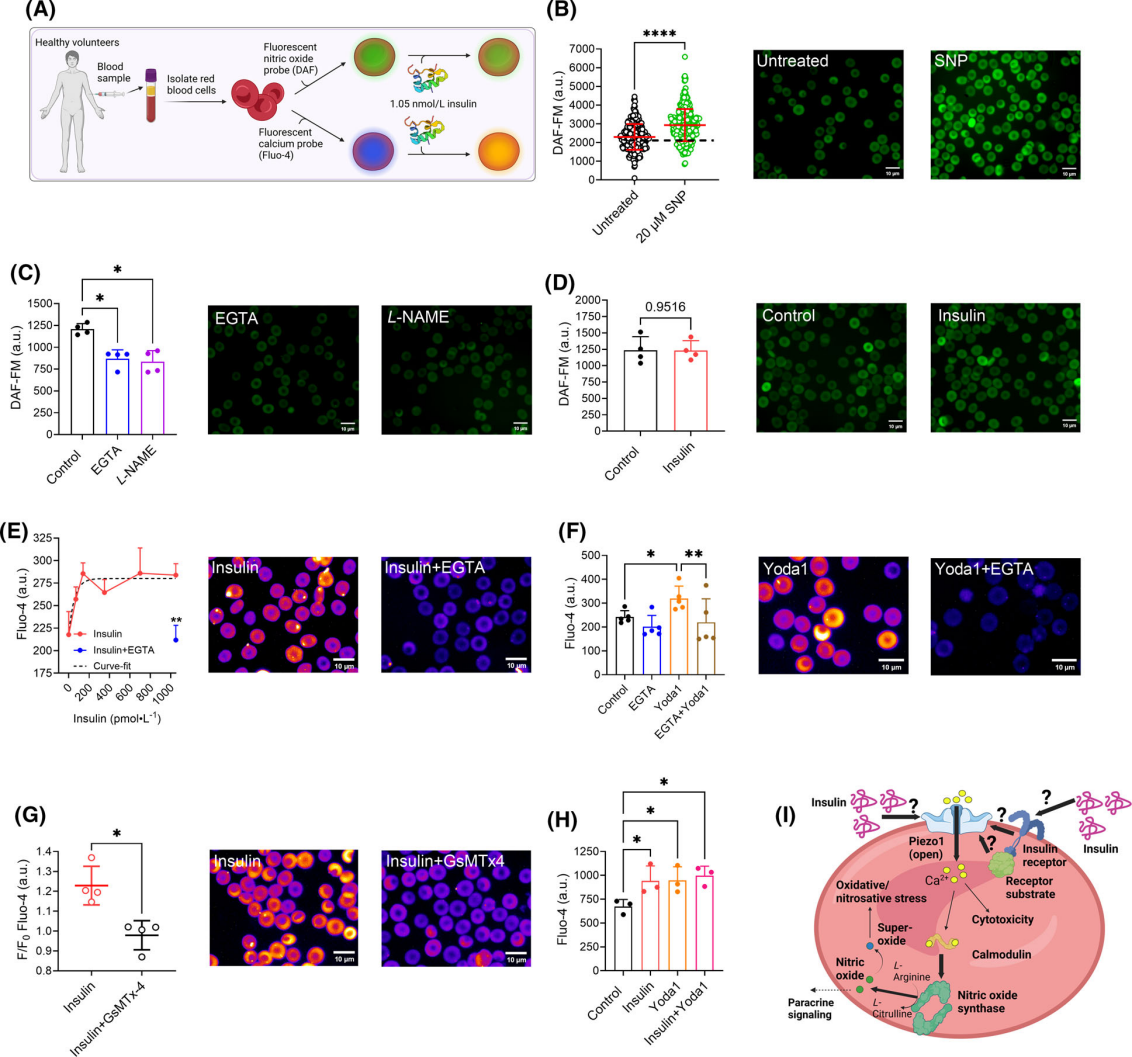
Human erythrocyte nitric oxide and calcium‐ion signaling through Piezo1 in response to insulin. (A) Human red blood cells (RBC) were isolated from blood samples and then loaded with fluorescent probes specific to nitric oxide (NO; DAF‐FM) or calcium‐ions (Ca^2+^; Fluo‐4). (B) The NO‐donor sodium nitroprusside (SNP) was employed to validate that DAF‐FM was sensitive to increased NO within RBC; *n* > 300 individual cells obtained from a single blood sample. ****P < 0.0001. (C) Mean DAF‐FM signal was recorded from untreated RBC and compared with that of RBC pre‐incubated with 10 mmol·L^−1^ of the Ca^2+^‐chelator ethylene glycol‐bis (β‐aminoethyl ether)‐N,N,N′,N′‐tetraacetic acid (EGTA) or the NO synthase inhibitor Nω‐Nitro‐L‐arginine methyl ester hydrochloride (L‐NAME) as negative controls. (D) Mean DAF‐FM signal was then recorded in RBC stimulated with 1.05 nmol·L^−1^ insulin and compared with that of unstimulated control RBC. (E) Dose–response curves of human RBC Fluo‐4 fluorescence, reflective of cytosolic Ca^2+^‐content, were constructed following exposure to increasing concentrations of insulin (0.07–1.05 nmol·L^−1^). (F) Alterations of Fluo‐4 were then measured following incubation with 10 mmol·L^−1^ of EGTA and stimulation with the Piezo1‐specific agonist Yoda1 (15 nmol·L^−1^). (G) Changes in Fluo‐4 fluorescent intensity of human RBC were recorded in response to insulin stimulation (1.05 nmol·L^−1^) in absence or presence of 3.3 μmol·L^−1^ of the mechanosensitive ion channel inhibitor GsMTx‐4. (H) Changes in Fluo‐4 fluorescence were measured following simultaneous exposure to insulin and Yoda1. (I) A schematic of the possible mechanisms linking insulin signaling with Piezo1 is depicted. **P < 0.01, *P < 0.05 determined using Mann–Whitney test (B), One‐way ANOVA (C, F, H) or unpaired t‐tests (D, E, G). *n* = 3–5 biological replicates (blood samples obtained from distinct donors). 150–300 individual RBC were analyzed per sample. Scale bars = 10 μm. Error bars denote standard deviation.

Human RBC were isolated from the blood of healthy volunteers, loaded with the fluorescent probes DAF‐FM or Fluo‐4, and then exposed to 1.05 nmol·L^−1^ of exogenous insulin (Fig. 4A). DAF‐FM fluorescence intensity of human RBC significantly increased following exposure to the NO‐donor SNP (*P <* 0.0001; Fig. 4B). In contrast, incubation with the Ca^2+^‐chelator EGTA or the NO synthase inhibitor *L*‐NAME significantly decreased DAF‐FM fluorescence intensity (both *P <* 0.05; Fig. 4C). Incubation of RBC with insulin did not affect DAF‐FM fluorescence intensity (*P =* 0.95; Fig. 4D).

### Insulin stimulates the uptake of calcium ions into human erythrocytes in a Piezo1‐dependent manner

Exposure of isolated human RBC to increasing concentrations of insulin resulted in accumulation of cytosolic Ca^2+^; incubation of RBC with EGTA prior to exposure to the highest concentration of insulin abolished the increase in cytosolic Ca^2+^ (*P <* 0.01; Fig. 4E). Increased cytosolic Ca^2+^ following exposure to insulin occurred in a dose‐dependent manner between 0.07 and 0.14 nmol·L^−1^, after which no further rise in [Ca^2+^]_i_ was observed. Non‐linear regression analysis yields a time constant of Τ = 0.055 nmol·L^−1^, indicating that 63.2% of the insulin‐stimulated Ca^2+^‐entry response occurs at this concentration. Exposure to the specific Piezo1‐agonist Yoda1 resulted in a comparable EGTA‐sensitive increase in cytosolic Ca^2+^ (both *P <* 0.05; Fig. 4F). Pre‐incubation with the non‐selective mechanosensitive channel blocker GsMTx‐4, however, prevented the increase in cytosolic Ca^2+^ induced by exposure to insulin (*P <* 0.05; Fig. 4G). When RBC were exposed to both Yoda1 and insulin simultaneously, a significant increase in Fluo‐4 fluorescence over that of control RBC was observed; however, no compounding effect was detected (all *P <* 0.05; Fig. 4H).

Collectively, it appears that insulin may stimulate Ca^2+^‐uptake of RBC via Piezo1, while not raising cytosolic NO (Fig. 4I). Given the profound relevance of a putative direct interaction potential crosstalk between insulin and Piezo1, we then performed *in silico* multimer modeling using AlphaFold 3 [26, 27] to determine whether direct insulin‐Piezo1 binding would be structurally feasible or whether insulin‐dependent Piezo1 activation is more likely transduced through other mechanisms.

### Insulin does not appear to interact directly with Piezo1

AlphaFold is used to predict structures of proteins based on their amino acid sequences (Table 2) with good accuracy, and AlphaFold‐multimer is an artificial intelligence algorithm trained on an extensive data set of homo‐ and heterodimers to predict potential binding sites in protein–protein interactions. While the structure of mouse Piezo1 (mPiezo1) has been solved and refined to a resolution of 3.46 Å [28], a recent report unveiled the structure of human Piezo1 (hPiezo1) for the first time, with an estimated resolution of ~3.3 Å [29]. The raw data are currently still under embargo; however, this is why we used the mPiezo1 structure here. Human insulin is released into the plasma as a hexamer, which then dissociates into dimers and monomers [30]; thus, we extracted the monomeric structure from an experimentally determined insulin hexamer structure (PDB 1AI0). We then used these experimentally determined mPiezo1 and insulin structures to validate the accuracy of the AlphaFold model (PDBs 6LQI, 1AI0; [31]).

**Table 2:** Amino acid sequences of the proteins used to model heterodimer in AlphaFold‐multimer.

Target gene	Amino acid sequence
PIEZO1 (human)	MEPHVLGAVLYWLLLPCALLAACLLRFSGLSLVYLLFLLLLPWFPGPTRCGLQGHTGRLLRALLGLSLLFLVAHLALQICLHIVPRLDQLLGPSCSRWETLSRHIGVTRLDLKDIPNAIRLVAPDLGILVVSSVCLGICGRLARNTRQSPHPRELDDDERDVDASPTAGLQEAATLAPTRRSRLAARFRVTAHWLLVAAGRVLAVTLLALAGIAHPSALSSVYLLLFLALCTWWACHFPISTRGFSRLCVAVGCFGAGHLICLYCYQMPLAQALLPPAGIWARVLGLKDFVGPTNCSSPHALVLNTGLDWPVYASPGVLLLLCYATASLRKLRAYRPSGQRKEAAKGYEARELELAELDQWPQERESDQHVVPTAPDTEADNCIVHELTGQSSVLRRPVRPKRAEPREASPLHSLGHLIMDQSYVCALIAMMVWSITYHSWLTFVLLLWACLIWTVRSRHQLAMLCSPCILLYGMTLCCLRYVWAMDLRPELPTTLGPVSLRQLGLEHTRYPCLDLGAMLLYTLTFWLLLRQFVKEKLLKWAESPAALTEVTVADTEPTRTQTLLQSLGELVKGVYAKYWIYVCAGMFIVVSFAGRLVVYKIVYMFLFLLCLTLFQVYYSLWRKLLKAFWWLVVAYTMLVLIAVYTFQFQDFPAYWRNLTGFTDEQLGDLGLEQFSVSELFSSILVPGFFLLACILQLHYFHRPFMQLTDMEHVSLPGTRLPRWAHRQDAVSGTPLLREEQQEHQQQQQEEEEEEEDSRDEGLGVATPHQATQVPEGAAKWGLVAERLLELAAGFSDVLSRVQVFLRRLLELHVFKLVALYTVWVALKEVSVMNLLLVVLWAFALPYPRFRPMASCLSTVWTCVIIVCKMLYQLKVVNPQEYSSNCTEPFPNSTNLLPTEISQSLLYRGPVDPANWFGVRKGFPNLGYIQNHLQVLLLLVFEAIVYRRQEHYRRQHQLAPLPAQAVFASGTRQQLDQDLLGCLKYFINFFFYKFGLEICFLMAVNVIGQRMNFLVTLHGCWLVAILTRRHRQAIARLWPNYCLFLALFLLYQYLLCLGMPPALCIDYPWRWSRAVPMNSALIKWLYLPDFFRAPNSTNLISDFLLLLCASQQWQVFSAERTEEWQRMAGVNTDRLEPLRGEPNPVPNFIHCRSYLDMLKVAVFRYLFWLVLVVVFVTGATRISIFGLGYLLACFYLLLFGTALLQRDTRARLVLWDCLILYNVTVIISKNMLSLLACVFVEQMQTGFCWVIQLFSLVCTVKGYYDPKEMMDRDQDCLLPVEEAGIIWDSVCFFFLLLQRRVFLSHYYLHVRADLQATALLASRGFALYNAANLKSIDFHRRIEEKSLAQLKRQMERIRAKQEKHRQGRVDRSRPQDTLGPKDPGLEPGPDSPGGSSPPRRQWWRPWLDHATVIHSGDYFLFESDSEEEEEAVPEDPRPSAQSAFQLAYQAWVTNAQAVLRRRQQEQEQARQEQAGQLPTGGGPSQEVEPAEGPEEAAAGRSHVVQRVLSTAQFLWMLGQALVDELTRWLQEFTRHHGTMSDVLRAERYLLTQELLQGGEVHRGVLDQLYTSQAEATLPGPTEAPNAPSTVSSGLGAEEPLSSMTDDMGSPLSTGYHTRSGSEEAVTDPGEREAGASLYQGLMRTASELLLDRRLRIPELEEAELFAEGQGRALRLLRAVYQCVAAHSELLCYFIIILNHMVTASAGSLVLPVLVFLWAMLSIPRPSKRFWMTAIVFTEIAVVVKYLFQFGFFPWNSHVVLRRYENKPYFPPRILGLEKTDGYIKYDLVQLMALFFHRSQLLCYGLWDHEEDSPSKEHDKSGEEEQGAEEGPGVPAATTEDHIQVEARVGPTDGTPEPQVELRPRDTRRISLRFRRRKKEGPARKGAAAIEAEDREEEEGEEEKEAPTGREKRPSRSGGRVRAAGRRLQGFCLSLAQGTYRPLRRFFHDILHTKYRAATDVYALMFLADVVDFIIIIFGFWAFGKHSAATDITSSLSDDQVPEAFLVMLLIQFSTMVVDRALYLRKTVLGKLAFQVALVLAIHLWMFFILPAVTERMFNQNVVAQLWYFVKCIYFALSAYQIRCGYPTRILGNFLTKKYNHLNLFLFQGFRLVPFLVELRAVMDWVWTDTTLSLSSWMCVEDIYANIFIIKCSRETEKKYPQPKGQKKKKIVKYGMGGLIILFLIAIIWFPLLFMSLVRSVVGVVNQPIDVTVTLKLGGYEPLFTMSAQQPSIIPFTAQAYEELSRQFDPQPLAMQFISQYSPEDIVTAQIEGSSGALWRISPPSRAQMKRELYNGTADITLRFTWNFQRDLAKGGTVEYANEKHMLALAPNSTARRQLASLLEGTSDQSVVIPNLFPKYIRAPNGPEANPVKQLQPNEEADYLGVRIQLRREQGAGATGFLEWWVIELQECRTDCNLLPMVIFSDKVSPPSLGFLAGYGIMGLYVSIVLVIGKFVRGFFSEISHSIMFEELPCVDRILKLCQDIFLVRETRELELEEELYAKLIFLYRSPETMIKWTREKE
INS (human)	Chain A: GIVEQCCTSICSLYQLENYCN Chain B: FVNQHLCGSHLVEALYLVCGERGFFYTPKT

**Fig. 5:**
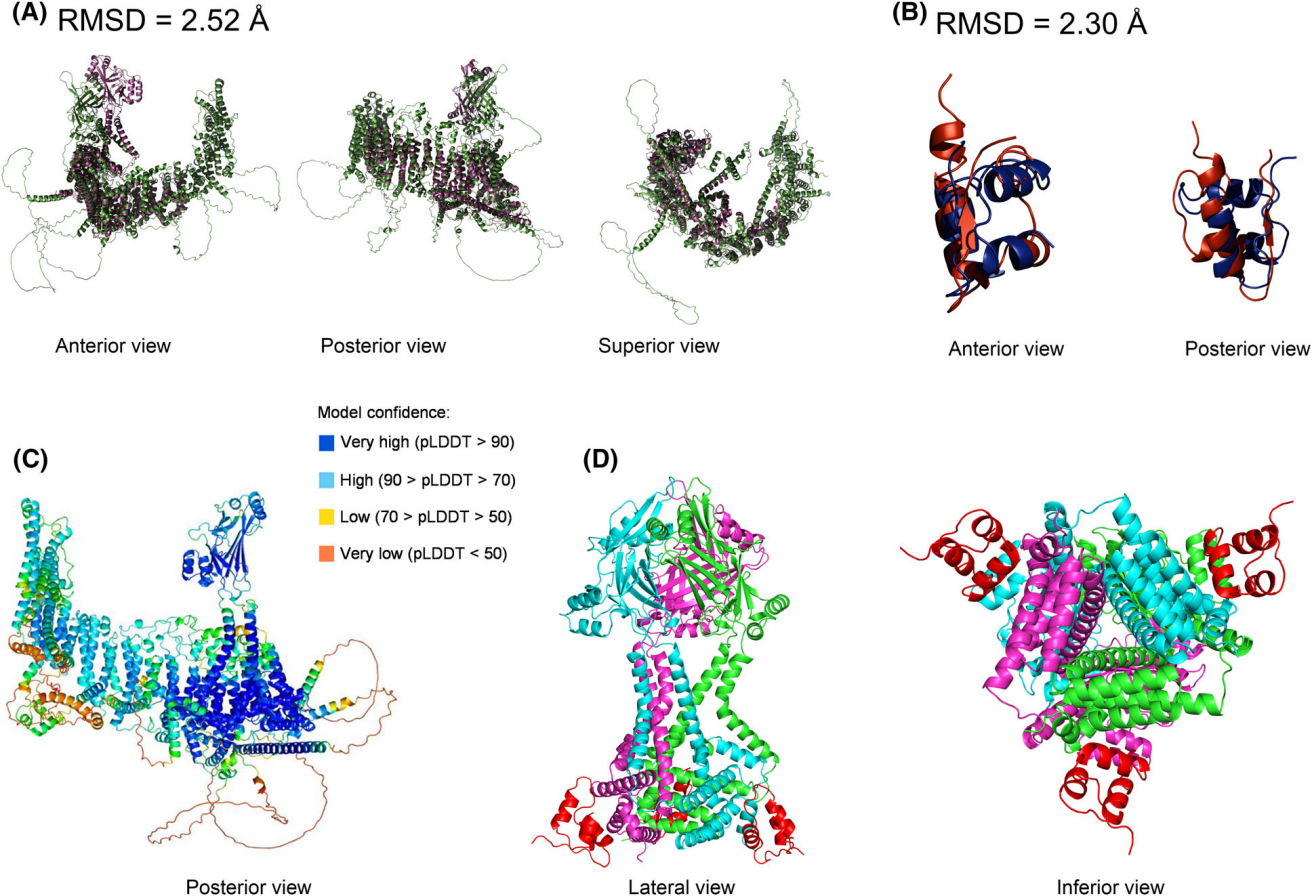
*In silico* modeling of the potential interaction between human Piezo1 and human insulin molecules. (A, B) The AlphaFold‐generated human Piezo1 and human insulin models were superimposed on experimentally obtained structures of mouse Piezo1 and human insulin, respectively, to validate structural accuracy. (C) The most highly ranked AlphaFold‐generated heterodimer of Piezo1 and insulin was colored in accordance with its corresponding confidence scores. (D) hPiezo1‐pore trimers with 3 insulin monomers are colored in magenta (chain A), green (chain B) and indigo (chain C), and insulin molecules are shown in red.

Next, we employed the amino acid sequences of hPiezo1 monomers and human insulin monomers to determine whether AlphaFold would predict any direct interaction (Fig. 5A). Superimposition of the experimental structures and AlphaFold‐generated model structures revealed a satisfactory degree of alignment for both Piezo1 (root mean square deviation RMSD = 2.52 Å; Fig. 5A) and insulin (RMSD = 2.30 Å; Fig. 5B). Modeling a potential interaction using the full sequence of one molecule of Piezo1 and one molecule of insulin, respectively, yielded strong heterodimer model confidence (pLDTT‐70‐90 for most residues; Fig. 5C), but no predicted binding. The maximum protein size for the AlphaFold algorithm is 2500 amino acids; thus, it is unable to accommodate modeling Piezo1 trimers, since three mPiezo1 molecules comprise a total of 7641 amino acids. The putative ion pore module of Piezo1 has been shown to be sufficient for enabling mechanosensitivity [32] and also harbors the binding sites for MDFIC, an auxiliary regulator of Piezo1 [33], and Yoda1, the pharmacological agonist of Piezo1 [34]. We therefore modeled a trimer of the hPiezo1 pore region (residues G1937‐E2521) to assess whether direct binding to insulin is structurally feasible (Fig. 5D). Collectively, we find that insulin is unlikely to directly interact with hPiezo1 monomers or trimers.

## Discussion

Employing a blend of *in vivo*, *in vitro*, and *in silico* techniques, we provide evidence to support a novel mechanism by which the anabolic hormone insulin facilitates entry of Ca^2+^ into human and murine RBC via the mechanosensitive cation channel Piezo1. Further, we report that this signaling pathway is pathologically altered by T2DM, which is characterized by hyperglycemia, obesity, and insulin resistance [1]. Under these conditions, we show that RBC are insensitive to insulin‐dependent Ca^2+^‐entry. Despite extensive research efforts, the increased CVD incidence in individuals with T2DM remains the most significant contributor to T2DM‐associated mortality and cannot be explained solely by blood vessel damage [3]. The hematological, biochemical, and functional alterations of RBC in this metabolically dysregulated environment likely contribute to the circulatory deficiencies observed in T2DM patients, and thus represent a novel risk factor that requires attention. The pathophysiological relevance of RBC signaling in chronic disorders of the cardiovascular system is still emerging [10]; however, RBC also provide an opportunity for entirely novel therapeutic strategies by providing previously disregarded molecular targets.

The traditional view of RBC depicts a biologically inert cell type with limited capacity for cellular signaling due to the lack of a nucleus and other subcellular structures; however, recent reports indicate that RBC do possess complex biochemical signaling processes with physiological relevance [10, 19, 35, 36]. In the current study, we observed that STZ‐injected mice fed a high‐fat diet for 15 week exhibited significantly elevated RBC [Ca^2+^]_i_ and markers of oxidative stress (Figs 1, 3). Conversely, these alterations did not precipitate abnormal RBC mechanics despite profound alterations in hematological parameters (Table 1) when assessed at a whole cell population level (Fig. 2), which are commonly observed in RBC obtained from T2DM patients [37] and postulated to contribute to microcirculatory dysfunction [38, 39]; however, the increased variability of membrane rigidity among individual RBC of the T2DM population (Fig. 2F) may indicate shifts that are not detectable on a cell population level. Altered hematological parameters of blood from T2DM mice included increased RBC count, hematocrit, and mean cell volume, collectively suggesting enhanced erythropoiesis, possibly stimulated by accelerated RBC turnover. The increase in hematocrit could impact the magnitude of shear forces generated within blood, which may have potentiating effects on Piezo1 activation long‐term, thus contributing to the accumulation of [Ca^2+^]_i_ in T2DM (Fig. 3). Shifts in the heterogeneous RBC population may be related to enhanced RBC removal through Ca^2+^‐pathways including Piezo1 [40, 41], which has been reported in rodent models of T2DM [42]; however, specific studies investigating RBC subpopulation dynamics in T2DM are required to ascertain this hypothesis.

An emerging body of evidence suggests that RBC drive cardiovascular complications in T2DM [10] due to a disturbance in oxidative balance [16, 17] leading to scavenging and depletion of RBC‐derived NO [15] that would otherwise contribute to the vascular NO pool [19]; instead, RBC‐derived NO is oxidized to the cytotoxic compound peroxynitrite (ONOO^−^) by superoxide (O_2_^−^). Given the rapid reaction time of NO and O_2_^−^ (<1 ms; [43]), intracellular accumulation of reactive oxygen species such as O_2_^−^ promotes scavenging NOS‐produced NO prior to its export from RBC. In agreement with previous reports supporting the presence of oxidative stress in this T2DM rodent model [44, 45, 46] and also in RBC from T2DM patients [47, 48, 49, 50, 51], we observed increased oxidative stress markers and DAF‐FM fluorescence in RBC obtained from T2DM mice (Figs 2, 3). DAF‐FM is a fluorescent probe that reacts with biochemical products capable of nitrosating reactions, including NO, but also ROS [52]. It is possible that the excess NO, should it escape Hb‐scavenging within RBC, caused the observed decrease in platelet activation markers in our study (Table 1). Nevertheless, further studies employing advanced analytical chemistry techniques with the capacity to discern the molecular composition of different oxidative and nitrosative species (e.g., spin trapping), including other RBC‐derived pools of NO (e.g., nitrosylated and nitrated proteins, plasma concentration of nitrite and nitrate), are warranted.

RBC obtained from T2DM mice exhibited increased basal cytosolic Ca^2+^‐content [Ca^2+^]_i_ (Fig. 3D,E). Maintenance of low [Ca^2+^]_i_ is critical for maintenance of RBC homeostasis [53], and conversely, elevated basal RBC [Ca^2+^]_i_ is associated with anemia [54], impaired cellular mechanics [55], osmotic fragility [56], and accelerated cell aging [57]. When exposed to exogenous insulin, RBC from control mice responded with an acute increase in [Ca^2+^]_i_, while RBC from T2DM mice were entirely unresponsive (Fig. 3F), despite relatively mild changes in metabolic T2DM markers (Fig. 1). While insulin resistance is a well‐described adaptation of various cell types in T2DM [58], it has not previously been reported in RBC, and the effects of insulin on RBC remain mysterious. It is possible that a loss of sensitivity to insulin‐stimulated Ca^2+^‐entry via Piezo1‐channels is due to a decrease in the number of channels on the RBC membrane. It appears that T2DM‐RBC samples presented with extracellular vesicles that had a strong Fluo‐4 fluorescence signal, which may have been released from RBC. Extracellular vesicles derived from mature RBC have been shown to contain various membrane proteins including Piezo1 [59] and age‐dependent loss of RBC membrane reportedly occurs in tandem with a loss of Piezo1 [40].

In contrast, RBC from neither group exhibited increased DAF‐FM fluorescence when exposed to exogenous insulin (Fig. 3C). Further, neither cytosolic NO concentration nor [Ca^2+^]_i_ was significantly associated with metabolic markers of diabetic severity (i.e., HbA1c and fasting blood glucose concentration). Enzymatic production of NO via NOS in RBC depends on an acute increase in [Ca^2+^]_i_ [20, 60]; however, it appears that the acute insulin‐dependent increase is insufficient to stimulate NOS activation, at least in our hands. Previous reports have indicated that acute insulin exposure may stimulate NOS activation [13] and NO production in RBC [24]; however, the insulin concentration used was either much higher than the physiological levels employed in the present study [24] or the incubation time was longer [13], which may explain the discrepancy in results.

The usefulness of animal models, including small mammals, for the study of human disease is indisputable; however, there are fundamental differences between the physiology of mice and man which limit the translatability of findings derived from these models [61]. Thus, we confirmed that the acute effects of exogenous insulin exposure hold true in human RBC, wherein acute insulin exposure did not facilitate RBC‐NO production, despite the uptake of Ca^2+^ (Fig. 3). Further, the chronically elevated basal [Ca^2+^]_i_ observed in RBC from T2DM mice was comparable to the level of [Ca^2+^]_i_ observed in insulin‐stimulated RBC from control mice. Given that RBC from T2DM patients retain normal Ca^2+^‐extrusion capacity, excess influx through overactivation of ion channels is the more probable cause for the accumulation of cytosolic Ca^2+^ [62]. It is plausible that the chronically elevated basal [Ca^2+^]_i_ lowers the additional Ca^2+^ required for NOS activation and NO production. Consequently, T2DM RBC produce excessive amounts of NO when additional Ca^2+^ accumulates in the cytosol due to, for example, activation of mechanosensitive ion channels during capillary transit [63]. Excess NO rapidly combines with superoxide (O_2_^−^) to form the cytotoxic molecule ONOO^−^, contributing to the redox imbalance observed in T2DM RBC [15, 17], which may ultimately contribute to endothelial dysfunction [10]. A limitation of the present study is that a murine model of T2DM was used, and detailed studies using RBC obtained from humans with T2DM will be essential to validating the present findings.

The mechanosensitive cation channel Piezo1 is expressed in RBC at ~80–200 copies per cell [64] and appears to regulate cell volume [55, 65], circulatory longevity [66], and NOS‐dependent NO production in response to shear stress (e.g., during cellular stretch in capillary transit [20, 63]). Pharmacological activation of Piezo1 by synthetic small molecule agonists (e.g., Yoda1, Jedi‐compounds [34, 67, 68]) is well described. Further, an auxiliary regulatory protein forming a complex with Piezo1 is known [33]; however, whether biochemical agonism of Piezo1 also occurs with physiologically relevant molecules remains to be elucidated. Here, we provide evidence of crosstalk between RBC insulin‐ and Piezo1‐signaling. Given the structural incompatibility of insulin and Piezo1 (Fig. 5), it seems unlikely that insulin could directly interact with Piezo1; however, insulin is known to undergo dynamic restructuring when engaging the insulin receptor, including partial refolding [69], the dynamics of which are likely not reproduced accurately in AlphaFold, and thus structural inference from this model should be interpreted with caution. Nonetheless, we hypothesize that downstream signaling initiated by the binding of insulin to its receptor is more likely to modulate gating of Piezo1 indirectly. RBC are known to express insulin receptors [70], which reportedly localize in the molecular neighborhood of Piezo1 [71]. Mutations in the *PIEZO1* gene induce xerocytosis/stomatocytosis (i.e., RBC dehydration) due to increased RBC [Ca^2+^]_i_, principally caused by prolonged Piezo1 channel inactivation kinetics [72, 73, 74]—a phenotype similar to that observed in the RBC‐T2DM in the present study, which may be caused by upregulation of Piezo1 activity by insulin signaling.

Collectively, the salient findings of the current study indicate that T2DM development in a murine model leads to pathological accumulation of Ca^2+^ and NO within RBC (Fig. 2). Given the prominence of free radicals within T2DM RBC, excess NO precipitates nitrosative stress, rather than providing beneficial effects. It appears that insulin resistance, a hallmark of T2DM, may also extend to RBC, following accumulation of [Ca^2+^]_i_. While the physiological function of this interaction remains elusive, elevated [Ca^2+^]_i_ is considered detrimental to normal RBC function [53], and circulatory deficits observed in T2DM patients may relate to insulin‐dependent overload of RBC [Ca^2+^]_i_.

## Materials and methods

### Experimental animals and T2DM model

A non‐genetic model of adult‐onset T2DM was employed in the present study, the details of which are described elsewhere [25]. The animal studies described herein were approved by the Griffith University Animals Ethics Committee (Ref no. PAM/05/22/AEC) and conform to the Australian Code of Practice for the Care and Use of animals for scientific purposes. Male 6‐week old C57Bl6/J mice (Animal Resources Centre Ltd., Canning Vale, WA, Australia) were housed at four animals per cage with access to food and water *ad libitum*. Mice were kept in a 12‐h day/night cycle at constant temperature (21 °C) and humidity (40%). All animals were initially fed a standard rodent chow. Following an acclimatization period of 2 week, mice were randomly allocated to control (*n* = 12) or T2DM (*n* = 12) groups. Due to temporal constraints imposed by *ex vivo* aging of RBC, not every measurement was performed on every individual blood sample; the number of biological replicates (i.e., samples from different mice on which measurements were performed) is indicated in each figure.

Mice in the T2DM phenotype group received a single dose of streptozotocin (STZ; 75 mg kg^−1^) to stress but not eliminate pancreatic β‐cells. STZ was administered via intraperitoneal injection at 8 week of age, following which the T2DM group was fed a high‐fat diet (HFD: 43.2% fats, 39.7% carbohydrates and 17.1% protein) for 15 week (Specialty Feeds, WA, Australia). The combination of STZ treatment and HFD feeding causes mice to develop an adult‐onset phenotype of T2DM characterized by hyperglycemia, hyperinsulinemia, impaired glucose clearance, and diminished circulatory function ([25]; ‘T2DM group’). Control mice received a single injection of sodium citrate in lieu of STZ and were fed standard rodent chow (18% fats, 63% carbohydrates, 19% protein). Unless otherwise indicated, chemicals were sourced from Sigma‐Aldrich (Castle Hill, NSW, Australia).

### Human and murine blood sampling

For murine blood, samples used for measurements of oxidative stress markers, NO‐ and Ca^2+^‐content were extracted via puncture of the submandibular vein from mice aged 23–25 week and collected into containers coated with 1.8 mg·mL^−1^ ethylenediaminetetraacetic acid (EDTA) for anticoagulation.

For human blood samples, five apparently healthy male volunteers aged 18–45 years were recruited for participation in the current study. Volunteers were recreationally active and presented with a body mass index <30 kg·m^−2^. The experimental procedures were approved by the Griffith University Ethics Committee (Ref no. 2020/093) and conform with the Declaration of Helsinki. Witnessed informed written consent was obtained and 10 mL of blood was sampled from a prominent vein in the antecubital region of the forearm. Blood was collected within 90 s of tourniquet application, in line with the international hemorheological guidelines [75] and immediately transferred into vacuum‐sealed tubes coated with 1.8 mg·mL^−1^ EDTA for anticoagulation. Blood samples used in this study were collected between February 2023 and February 2024 at Griffith University, Gold Coast Campus, Queensland, Australia.

### Isolation of red blood cells

Human or murine whole blood was centrifuged at 1500 ***g*** for 10 min to separate plasma and cellular constituents. The buffy coat and plasma were removed, and the RBC pellet was then washed three times using PBS. For RBC intended to be stained with Fluo‐4, RBC were washed three times using Tyrode's buffer instead.

### Metabolic tests

Body mass of mice was determined weekly using electronic laboratory scales. Glucose tolerance tests (GTTs) were performed in all mice 15 week post‐injection to assess glucose clearance capacity. GTTs were performed following a 4‐h fasting period by administering a bolus of glucose at 2 g·kg^−1^ via intraperitoneal injection, following which blood samples were collected from the tail tip at 0, 30, 60, 90, 120, and 180 min post‐injection. Blood glucose concentration was then measured using a glucometer (Accu‐check II, Roche Diagnostics, Castle Hill, Australia). Glycated hemoglobin (HbA1c) was measured using the Mouse Hemoglobin A1c ELISA Kit (ab285317; Abcam, Melbourne, VIC, Australia) according to the manufacturer's instructions.

### Hematological analyses

Blood cell counts and hematological analyses were performed on an automated cell counter (DxH 500, Beckman Coulter Inc., Brea, CA, Australia) operated according to the manufacturer's recommendations. Microcytic anemia factor (MAf), a parameter derived from RBC mean cell volume (MCV) and total hemoglobin concentration (Hb) that is sensitive to clinical anemic conditions and indicative of accelerated RBC turnover [76], was calculated as:

$$\mathrm{MAf}=\frac{\left(\mathrm{MCV}\times \mathrm{Hb}\right)}{100}$$

### Cytosolic reactive oxygen and nitrogen species assay

Cytosolic ROS/RNS of RBC obtained from healthy and T2DM mice were measured using a commercially available assay (OxiSelect ROS/RNS, Cell Biolabs Inc., San Diego, CA, USA), in accordance with the manufacturer's instructions. Briefly, the assay employs a proprietary fluorescent probe that is structurally similar to 2′,7′‐dichlorodihydrofluorescein diacetate, a popular detection molecule sensitive to both ROS and RNS [77]. A standard curve of increasing concentrations of hydrogen peroxide is constructed, and the read‐out fluorescent intensity is proportional to ROS/RNS concentration. Fluorescent intensity was assessed using a fluorometric plate reader (FLUOstar Omega, BMG Labtech, Mornington, VIC, Australia) with filters set to excitation/emission wavelengths of λ = 480 nm and λ = 518 nm, respectively. Concurrently, total Hb of a given sample was determined using the Harboe method [78]. Briefly, this spectrophotometric method exploits detection of the Soret band of Hb at λ = 415 nm (FLUOstar Omega, BMG Labtech, Mornington, VIC, Australia) and employs a correction based on absorbance measurements at λ = 380 and 450 nm. Total ROS/RNS concentration was calculated and then normalized to the total Hb content of a given sample.

### Cytosolic nitric oxide detection

Isolated RBC were loaded with 5 μmol L^−1^ diaminofluorescein‐FM diacetate (DAF‐FM DA) under constant gentle agitation on a laboratory tube roller for 30 min at room temperature. Following loading with DAF‐FM, the RBC pellet was washed with 0.1 m phosphate buffered saline (PBS; adjusted to 290 ± 5 mOsm, pH 7.4 ± 0.05) three times and then resuspended in a modified physiological Tyrode's buffering solution containing (in mmol L^−1^): 135 NaCl, 5.4 KCl, 10 glucose, 1 MgCl_2_, 1.8 CaCl_2_, and 10 HEPES (pH 7.35), supplemented with 0.0001 L·L^−1^ BSA and 1 mmol·L^−1^ L‐Arginine. Resuspended RBC were then incubated at 37 °C for 15 min, with an additional aliquot prepared and incubated concurrently in the presence of 1.05 nmol·L^−1^ (= 175 μIU·mL^−1^) insulin, and placed on a cover slip for imaging.

### Cytosolic calcium‐ion detection

Isolated RBC were washed thrice in Tyrode's buffer. Washed RBC were then loaded with 5 μmol L^−1^ Fluo‐4 AM under constant gentle agitation on a laboratory tube roller for 60 min at room temperature. Following loading with Fluo‐4, the RBC pellet was washed thrice in Tyrode's buffer and then resuspended in the same buffer supplemented with 0.0001 L·L^−1^ BSA. Resuspended RBC were incubated at 37 °C for 15 min, with an additional aliquot prepared and incubated concurrently in the presence of 1.05 nmol·L^−1^ insulin and then placed on a cover slip for imaging.

### Red blood cell deformability and shear modulus measurements

Mechanical properties of RBC were assessed at both population and single cell levels. A commercial ektacytometer (LORCA MaxSis, RR Mechatronics, de Hoorn, The Netherlands) was employed to assess the deformation of RBC under well‐controlled mechanical forces. This instrument is described in detail elsewhere [79]; in brief, the ektacytometer comprises a rotatable cup separated from a static inner bob by a narrow gap (~300 μm). The blood sample is diluted in a highly viscous isosmotic polyvinylpyrrolidone medium (28.1 mPa s, 288 mOsm, pH 7.41) and suspended into the gap. Precise control over cup rotation velocity enables the application of well‐controlled shear stresses onto the blood sample; blood is exposed to a ramped protocol of increasing shears, while laser diffraction patterns indicative of RBC deformation generated by emittance through the sheared sample are recorded simultaneously. An ellipse is fit to the diffraction patterns and an elongation index is calculated using the length and width of the ellipse (EI = (length − width)/(length + width)).

The shear modulus of individual RBC was measured using a custom‐built micropipette aspiration apparatus, which is described in detail elsewhere [80]. Blood was centrifuged at 1500 ***g*** for 10 min to separate plasma and cellular constituents. Plasma and buffy coat were removed, and RBC were then washed twice with PBS. Washed and isolated RBC were resuspended at 0.001 L·L^−1^ hematocrit in PBS supplemented with 0.01% w·v^−1^ BSA for micropipette assessments. Using borosilicate glass micropipettes with an inner diameter of 1 μm, individual RBC were investigated with stepwise increases in aspiration pressure (0, 13, 36, 75 Pa), which were monitored in real‐time using a pressure sensor and DAQ system (MLT0380/A; PowerLab, ADInstruments Pty Ltd, NSW, Australia). Aspiration with this pressure range enabled measurement of a cell's shear elastic modulus by measuring the aspirated portion of the cell (L_p_) at each pressure increment (ΔP). To standardize this measurement for comparison, L_p_ was subsequently normalized for the radius of the pipette tip (r_p_), and suction force (pN) was determined by calculating the ΔP exerted across the area of the micropipette tip (π·r_p_^2^). The experimental determination of the slope of L_p_·r_p_^−1^ and suction force (pN) yields the membrane shear modulus (μ) for a single cell in units pN·μm^−1^.

### DAF‐FM loading and stimulation of nitric oxide synthase in human erythrocytes

Isolated human RBC were loaded with DAF‐FM DA using the same procedures described here for murine RBC. Isolated RBC were resuspended in Tyrode's buffer supplemented with 0.1 mL·L^−1^ BSA and 1 mmol·L^−1^ *L*‐Arginine. Resuspended RBC were incubated with the NO‐donor sodium nitroprusside (SNP; 20 μmol·L^−1^) as a positive control, while other aliquots were incubated with the Ca^2+^‐chelator ethylene glycol‐bis (β‐aminoethyl ether)‐N,N,N′,N′‐tetraacetic acid (EGTA; 10 mmol·L^−1^) or the NO synthase inhibitor Nω‐Nitro‐L‐arginine methyl ester hydrochloride (*L*‐NAME; 10 mmol·L^−1^) as negative controls. A separate aliquot of resuspended RBC was incubated with 1.05 nmol·L^−1^ insulin, while another aliquot was incubated without insulin. All incubations were performed at 37 °C for 15 min.

### Fluo‐4 loading and stimulation of Piezo1 in human erythrocytes

Isolated human RBC were loaded with Fluo‐4 AM using the same procedures described here for murine RBC. Isolated RBC were resuspended in Tyrode's buffer supplemented with 0.1 mL·L^−1^ BSA. Samples were incubated at 37 °C for 2 min. Extracellular Ca^2+^ was chelated in some conditions using 10 mmol·L^−1^ EGTA. Piezo1 stimulation was performed via exposure to 15 nmol·L^−1^ of the specific channel agonist Yoda1 [67]. Piezo1 inhibition in the presence of 1.05 nmol·L^−1^ insulin was performed via concurrent incubation with 3.3 μmol·L^−1^ of the non‐selective mechanosensitive channel blocker GsMTx‐4 [81].

### Image acquisition and analysis

Following dispersion of RBC loaded with fluorescent probes onto cover slips, they were incubated at room temperature for 15 min to allow for de‐esterification of the dyes and settling of RBC onto the imaging plane. Cells were then illuminated with an argon laser (excitation, λ = 488 nm; and emission, λ = 505 nm), and images were captured using an inverted fluorescence microscope (IX73, Olympus, Tokyo, Japan) with an integrated camera (optiMOS sCMOS, QImaging, Surrey, Australia) at 600‐fold magnification. Images were taken in both brightfield mode and under fluorescent illumination in at least three distinct areas per cover slip.

### Modeling of protein–protein interactions

The AlphaFold3 algorithm [27] was used to determine potential protein–protein interactions between human Piezo1 and human insulin via the AlphaFold server (https://www.alphafoldserver.com/). The amino acid sequences of human Piezo1 and human insulin were utilized for analysis (Table 2).

### Data analysis

Elongation index (EI) values recorded at specific shear stresses were fit using a non‐linear modification of the Lineweaver–Burk equation as previously described [82], wherein *R* ^2^ ≥ 0.98 was deemed an acceptable fit to facilitate accurate interpolation of EI values. Using the Lineweaver–Burk equation, parameters reflecting cellular deformability of RBC populations were calculated (maximal theoretical EI over infinite shear stress (EI_max_) and half the shear stress required for maximal deformation (SS_1/2_)). Shear modulus of individual RBC was calculated as reported previously [80].

RBC were loaded with the fluorescent probes DAF‐FM DA and Fluo‐4 to estimate intracellular concentrations of NO and Ca^2+^, respectively. In order to ensure measured effects were representative, mean fluorescent intensities of 150–300 individual RBC were calculated for a given sample, and blood samples from at least 9 different mice or at least 3 different human donors were analyzed. All RBC visible in images taken in at least 3 randomly chosen areas on the coverslip were included in the analysis, provided the entire cell area was visible and there was no overlap with other cells. Fluorescent intensity was determined using the open‐source analysis software FIJI (version 1.53c [83]; National Institutes of Health, Bethesda, MD, USA).

### Statistical analysis

Mean group differences in mouse body weight across the study were compared using a two‐way analysis of variance (ANOVA) with Bonferroni's *post*‐*hoc* test. Differences in glucose tolerance between control and T2DM mice were assessed using a two‐way ANOVA with Bonferroni's *post hoc* test. Differences in mean EI values at specific shear stresses were determined using a two‐way ANOVA with Tukey's *post hoc* test.

For group comparisons between healthy control mice and T2DM mice, data distribution was determined using the Shapiro–Wilk normality test, and differences in the means were then assessed using either unpaired *t*‐tests or the Mann–Whitney U‐test.

Differences in fluorescent intensity of human RBC treated with chemical agents were determined using one‐way ANOVAs when three or more groups were compared. Comparisons between two groups were carried out using unpaired *t*‐tests or Mann–Whitney U‐tests depending on the outcome of the Shapiro–Wilk normality test.

An α level of 0.05 was used to determine statistical significance; all data analyses and statistical tests were performed using commercially available software (Prism, GraphPad Software, release 9.20, San Diego, CA, USA). Data are presented as mean ± standard deviation, unless noted otherwise.

## Conflict of interest

The authors have no competing interests to report.

## Author contributions

LK, TAG, APM, JNP, AS, LAS, and MJS contributed to study design and conception. LK, TAG, APM, JNP, AS, LAS, EFD, and MJS acquired funding. TAG, JNP, JHW, AS, LAS, KR, and EFD developed the rodent model. LK, TAG, APM, JHW, and KR contributed to data collection. LK performed data analysis, prepared the figures, performed AlphaFold‐modeling, and wrote the first draft of the manuscript. All authors revised the manuscript for important intellectual content.

## Peer review

The peer review history for this article is available at https://www.webofscience.com/api/gateway/wos/peer‐review/10.1111/febs.70157.

## Data Availability

The datasets used and/or analyzed during the current study are available from the corresponding author on reasonable request.
